# The population attributable fraction of cases due to gatherings and groups with relevance to COVID-19 mitigation strategies

**DOI:** 10.1098/rstb.2020.0273

**Published:** 2021-07-19

**Authors:** Ellen Brooks-Pollock, Jonathan M. Read, Thomas House, Graham F. Medley, Matt J. Keeling, Leon Danon

**Affiliations:** ^1^ Bristol Veterinary School, University of Bristol, Bristol BS40 5DU, UK; ^2^ Population Health Sciences, Bristol Medical School, Bristol, BS8 2BN, UK; ^3^ Lancaster Medical School, Lancaster University, Lancaster LA1 4YW, UK; ^4^ Department of Mathematics, University of Manchester, Manchester M13 9PL, UK; ^5^ Centre for Mathematical Modelling of Infectious Disease, London School of Hygiene and Tropical Medicine, London WC1H 9SH, UK; ^6^ Mathematics Institute and Department of Life Sciences, University of Warwick, Coventry CV4 7AL, UK; ^7^ Department of Engineering Mathematics, University of Bristol, Bristol BS8 1UB, UK

**Keywords:** COVID-19, population attributable fraction (PAF), gatherings

## Abstract

Many countries have banned groups and gatherings as part of their response to the pandemic caused by the coronavirus, SARS-CoV-2. Although there are outbreak reports involving mass gatherings, the contribution to overall transmission is unknown. We used data from a survey of social contact behaviour that specifically asked about contact with groups to estimate the population attributable fraction (PAF) due to groups as the relative change in the basic reproduction number when groups are prevented. Groups of 50+ individuals accounted for 0.5% of reported contact events, and we estimate that the PAF due to groups of 50+ people is 5.4% (95% confidence interval 1.4%, 11.5%). The PAF due to groups of 20+ people is 18.9% (12.7%, 25.7%) and the PAF due to groups of 10+ is 25.2% (19.4%, 31.4%). Under normal circumstances with pre-COVID-19 contact patterns, large groups of individuals have a relatively small epidemiological impact; small- and medium-sized groups between 10 and 50 people have a larger impact on an epidemic.

This article is part of the theme issue ‘Modelling that shaped the early COVID-19 pandemic response in the UK’.

## 1. Introduction

Preventing social contacts has been used worldwide in 2020 to reduce transmission of the novel coronavirus, SARS-CoV-2. Early restrictions introduced sought to limit the number of people that could meet at one time. In the UK, Scotland banned all large gatherings over 500 people in March 2020, and gatherings were to be banned in England but then the national stay-at-home order was introduced on 23 March 2020. As the first lockdown was eased, gatherings or more than 30 were banned, which was then reduced to ‘the rule of six' in September 2020 to prevent groups of more than six individuals meeting simultaneously.

Given knowledge of transmission mechanisms, bringing together groups of people into the same space should prove conducive for the spread of close-contact infectious diseases. Indeed, gatherings have been associated with outbreaks of communicable diseases such as measles [1], influenza [2] and meningitis [3]. Public health agencies, including the World Health Organization, have specific guidance for preventing disease outbreaks at mass gatherings [4]. Factors such as the age of participant [1], zoonotic transmission and the presence of animals [5], crowding [6,7], the lack of sanitation [7] and location and event duration [6] are associated with the reporting of mass gathering-related outbreaks.

Despite the evidence of the importance of gatherings for disease transmission from intuition and individual outbreaks, the population-level impact of different mass gathering policies has not been established. While systematic reviews have identified outbreak reports involving mass gatherings [5,6], the overall impact of mass gatherings could not be quantitatively assessed. A detailed modelling study of disease transmission in the state of Georgia, USA, found that in extreme scenarios when 25% of the population participated in a 2-day long gathering shortly before the epidemic peak, peak prevalence could increase by up to 10%. More realistic scenarios resulted in minimal population-level changes [8].

The population attributable fraction (PAF) is a measure of the importance of a risk factor to disease burden or death in a population, borrowed from non-communicable disease epidemiology [9]. The PAF of a risk factor is the percentage of disease burden or mortality that can be attributed to the presence of that increased risk; an example is the PAF of lung cancer cases that are due to smoking. In previous work, we demonstrated that for infectious diseases, the PAF can be estimated as the percentage change in the basic reproduction number (average number of secondary cases per infectious case in an otherwise susceptible population [10]) in the counterfactual situation where the risk factor is removed from the population [11]. Here, we use representative data on individuals' daily social contacts, including group contacts, to estimate the PAF due to groups and gatherings.

## 2. Methods

### (a) Social contact data

The social contact survey (SCS) [12,13] collected data on social contacts from 5388 participants between 2009 and 2010 in the UK. Participants were asked to enumerate other people with whom they had had contact over the course of a single day. Contacts were defined as those with whom participants had a face-to-face conversation within 3 m and/or physically touched skin-on-skin. Participants were able to report individual contacts and up to five groups of contacts, for instance church groups, weddings, large work functions or multiple contacts at work. The ‘groups' question was designed to aid participants in reporting multiple similar contacts. Group contacts were defined in the same way as individual contacts, i.e. if a person attended a concert with 1000 people, but only spoke to five people, the number of recorded group contacts would be five. Participants were asked whether members of the group knew each other.

As well as the number of contacts, participants were asked to estimate the length of time spent with each contact or group of contacts as either: less than 10 min, 11–30 min, 31–60 min or over 60 min, the distance from home, the frequency with which the contact took place and whether it involved physical contact.

The SCS data are available to download at http://wrap.warwick.ac.uk/54273/.

### (b) Reproduction numbers and population attributable fraction

We calculate the basic reproduction number with and without groups of various sizes. For each participant *j*, we use their *j_k_* contact reports to calculate their individual reproduction number, $R_{\mathrm{ind}}^{j}$. We assumed that $R_{\mathrm{ind}}^{j}$ is proportional to the number of individuals reported in the contact, *n_i_*, (*n_i_* = 1 for single contacts, *n_i_* > 1 for groups) multiplied by the duration of each contact, *d_i_*:

2.1 $$R_{\mathrm{ind}}^{j}\propto \overset{\;j_{k}}{\underset{i=1}{\sum }}n_{i}d_{i}.$$

**Figure 1.**
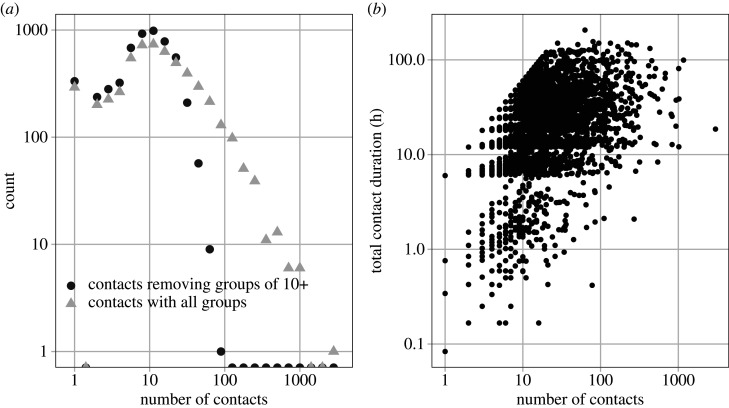
(*a*) The distribution in the number of social contacts per participants from the SCS (*n* = 5388) with and without groups of 10 and greater. Even without groups of 10 or more, individuals can have more than 10 other contacts. (*b*) The relationship between number of contacts and total contact duration.

The duration of each contact is taken as the mid-point of each time interval, i.e. 5 min, 20 min, 45 min and 6 h, as recorded by the participant. The interpretation of contact duration is different for individual versus group contacts, as there is a limit to the number of face-to-face contacts that one person can make in a finite time. We observe a saturation of contact duration for individuals with large numbers of contacts (figure 1*b*). The saturation occurs between 20 and 30 contacts per individual. We adjust for this by dividing the duration of group contacts by the number of individuals in the group, when the number of group contacts is greater than a random number between 20 and 30.

The population-level reproduction number is the average number of secondary cases caused by an average infectious person. Individuals with higher $R_{\mathrm{ind}}^{j}$ will contribute more to the population-level *R_t_* because they are more likely to get infected than individuals with lower $R_{\mathrm{ind}}^{j}$. Therefore, we estimate *R_t_* as a bootstrap resample (random sample with replacement) of the individual reproduction numbers weighted by the individual reproduction numbers:

2.2 $$R_{t}^{*}=\mathrm{Boot}({\left(R_{\mathrm{ind}}^{j}\right)}^{2})\;j=1,\;\ldots ,\;N,$$

where *N* is the number of participants in the SCS. The mean and 95% confidence interval (CI) were calculated from the bootstrapped sample.

We calculated the PAF for groups of size *G* or greater as the percentage change in the basic reproduction number:

$${\mathrm{PAF}}_{G}=1-R_{t}^{*}(\mathrm{without}\;\;\mathrm{groups}\geq G)/R_{t}^{*}(\mathrm{with}\;\;\mathrm{groups}).$$

We investigate the PAF for groups of greater than 10, and up to groups greater than 100, in increments of 10. We investigated differences between groups that knew each other and groups that did not know each other.

## 3. Results

### (a) Impact of groups on numbers of contacts per person

A total of 48 001 unique contacts were reported by 5388 participants. Of those, 42 945 (89%) were individual contacts and 5056 groups were reported (accounting for 11% of reported contacts). The median and mean number of contacts per person were 11.5 and 27.0, range 1 to 3011 (figure 1*a*).

Nearly half, 45%, of participants reported group contacts. The majority of groups reported (3860; 76%) were groups of people who knew each other; 2979 (59%) groups had 10 or fewer members; the median and mean reported group size was 9 and 20.3 individuals, respectively.

Restricting contacts to groups of size 50 or less reduces the median and mean number of individual contacts per person to 11.0 and 18.8; restricting contacts to groups of size 20 or less reduces the median and mean number of contacts per person to 10.0 and 14.1; restricting contacts to groups of size 10 or less reduces the median and mean number of contacts per person to 9 and 11.0. Figure 1 shows the degree distribution (number of contacts) per person with and without contacts associated with groups of size greater than 10.

Compared to individual contacts, group contacts were more likely to be more than 2 miles from the participants home (64% versus 51%), less likely to involve physical contact (25% versus 44%) and more likely to involve new individuals (21% versus 15%).

### (b) Population attributable fraction

**Figure 2.**
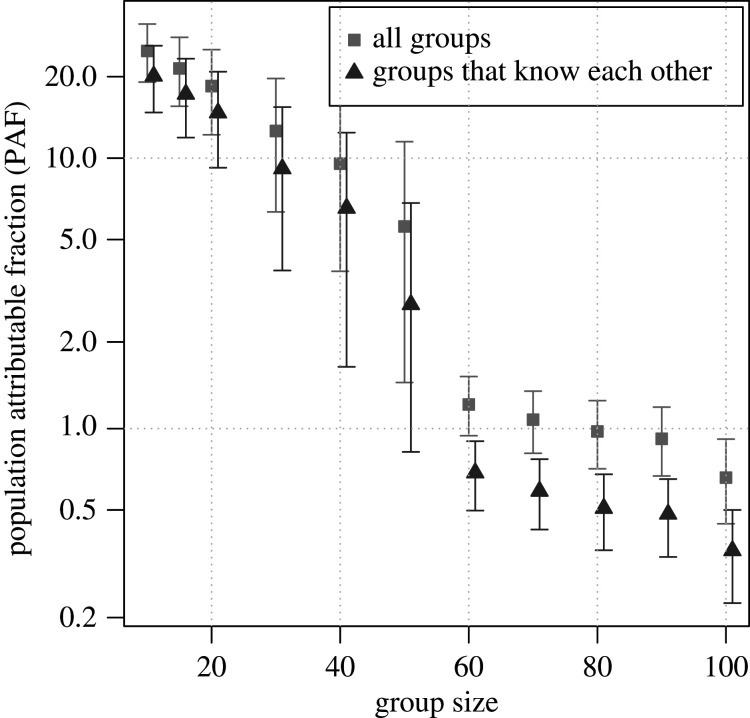
The PAF of cases due to groups of various sizes. The squares are all groups and the triangles are groups of people who are known to each other. The error bars are 95% confidence intervals.

The PAF due to groups decreased with increasing group size. For the largest groups with more than 100 individuals the PAF_100_ is estimated at 0.6% (95% CI: 0.4%, 0.8%). The PAF_50_ is estimated at 5.5% (95% CI: 1.4%, 11.4%); the PAF_20_ is 18.9% (95% CI: 12.7%, 25.7%); the PAF_10_ is 25.2% (95% CI: 19.4%, 31.4%) (figure 2).

The pattern of decreasing PAF with increasing group size is seen for both groups of individuals who are known to each other and groups of individuals who are unknown to each other. The PAF due to groups of 10+ known to each other is estimated at 20.6% (95% CI: 15.4%, 26.3%) and due to groups of 50+ known to each other is estimated at 2.9% (95% CI: 0.8%, 6.9%). The remaining contribution to *R_t_* is due to contact with individuals.

The estimated impact of large groups on *R_t_* is due to the relative frequency with which they are reported in the SCS, i.e. under normal (pre-COVID) contact patterns.

## 4. Discussion

In this paper, we analysed social contact data in the context of infectious disease transmission and gatherings. Our findings suggest that large groups of individuals have a relatively small impact on an epidemic, under the assumption that contact patterns remain otherwise constant. This is owing to the relative rarity of large-scale gatherings and the sub-linear scaling between number of contacts and infectivity.

The SCS is one of a number of social contact surveys that have been conducted to quantify the impact of social mixing on disease transmission [14–18]. The SCS specifically asked about groups of similar contacts. These groups are not necessarily public or mass gatherings and represented groups that both knew each other and those that did not. The group sizes reported in the SCS were not necessarily the same size of an event where contacts may have taken place. Therefore, this analysis should be considered in terms of contacts per person, rather than to guide the acceptable size of organized events. The SCS asked about contacts on a single day and did not capture multi-day events; simulation studies have shown that prolonged mass gatherings were necessary to alter the course of an epidemic [8]. Our analysis was based on social contact data collected between 2009 and 2010; contact patterns may have altered in the past decade. We also did not account for individuals changing their behaviour if group activities were cancelled.

In the context of COVID-19 mitigation, this analysis considered one aspect of gatherings: the impact on an epidemic. However, there may be other valid reasons for preventing mass events, such as policing and managing resources. Our analysis implicitly assumes that infection is already present in the population as is the case for COVID-19; for other diseases, gatherings can be associated with increased global travel which can bring new strains into an area or result in out-of-season outbreaks, which were also not captured here.

Our findings illustrate the difficult choices that are necessary to limit COVID-19 spread. Meetings of large groups of more than 100 individuals are relatively infrequent, and their prohibition may have a limited impact on the epidemic. More epidemiologically relevant are groups of 10 to 20 people, as they occur more frequently and could potentially have a larger impact on transmission; they may also involve inter-generational family groups. This analysis was designed to aid decision-making in the context of social distancing measures to control COVID-19 and should be considered against alternative control strategies so that the most effective measures can be implemented in the long term.

## In context

This analysis of the impact of gatherings was submitted to the Scientific Pandemic Influenza Group on Modelling on 11 March 2020. On that date, there had been 1297 confirmed cases and 25 deaths attributable to COVID-19 in the UK. The Italian government had imposed a national quarantine, or ‘lockdown', 2 days earlier and there were active discussions about the scale and type of social distancing measures that would be used in the UK.

Early restrictions sought to limit the number of people that could meet at one time. On 12 March 2020, the First Minister of Scotland announced that all large gatherings over 500 people would be cancelled from the following Monday, and the UK Prime Minister announced on 13 March 2020 that gatherings (of unspecified size) would be banned in England from the following weekend. As it transpired, by the following weekend, a national stay-at-home order was announced in the UK. However, as the lockdown was eased, attention turned back to the size of gatherings. An amendment to the Coronavirus Health Protection Regulation 2020 was made in August 2020 that restricted organizing or facilitating gatherings of more than 30 persons. On 14 September 2020, ‘the rule of six' was introduced that prevented groups of more than six individuals meeting simultaneously.

This paper was one piece of evidence that suggested that minimal social distancing measures, such as banning gatherings on their own, would not be sufficient to prevent a large-scale epidemic in the UK. When we first conducted this analysis in March 2020, we calculated the population-level reproduction number as the unweighted mean of the individual reproduction numbers, which led to smaller attributable fractions to groups. Ideally, we would have constructed a who-infected-whom matrix, but the SCS did not contain the age of contacts. Instead, to capture the fact that highly connected individuals contribute more to the reproduction number than less well-connected individuals, we used the individual reproduction numbers as a weight with which to calculate a weighted mean of reproduction numbers.

This analysis was conducted before genomic sequencing could be used to quantify the role of superspreading and large events. In a phylogenetic analysis of cases in the Boston area, USA [19], 29% of cases were reported to be responsible for 85% of secondary infections. In our analysis, 29% of participants reported 77% contacts. After accounting for contact duration and risk of infection (as we do in the paper), 29% of individuals contribute 84% of secondary cases, suggesting that using social contact data is able to capture the overdispersion of superspreading. Other features of large events, such as the increased distanced travelled to group contacts, are not captured in our analysis.

## Data accessibility

The SCS data are available to download at http://wrap.warwick.ac.uk/54273/.
